# Functional Organisation of the Mouse Superior Colliculus

**DOI:** 10.3389/fncir.2022.792959

**Published:** 2022-04-29

**Authors:** Thomas Wheatcroft, Aman B. Saleem, Samuel G. Solomon

**Affiliations:** Institute of Behavioural Neuroscience, University College London, London, United Kingdom

**Keywords:** mouse vision, instinctive behaviour, midbrain, threat, approach, sensorimotor

## Abstract

The superior colliculus (SC) is a highly conserved area of the mammalian midbrain that is widely implicated in the organisation and control of behaviour. SC receives input from a large number of brain areas, and provides outputs to a large number of areas. The convergence and divergence of anatomical connections with different areas and systems provides challenges for understanding how SC contributes to behaviour. Recent work in mouse has provided large anatomical datasets, and a wealth of new data from experiments that identify and manipulate different cells within SC, and their inputs and outputs, during simple behaviours. These data offer an opportunity to better understand the roles that SC plays in these behaviours. However, some of the observations appear, at first sight, to be contradictory. Here we review this recent work and hypothesise a simple framework which can capture the observations, that requires only a small change to previous models. Specifically, the functional organisation of SC can be explained by supposing that three largely distinct circuits support three largely distinct classes of simple behaviours–arrest, turning towards, and the triggering of escape or capture. These behaviours are hypothesised to be supported by the optic, intermediate and deep layers, respectively.

## The Superior Colliculus

The superior colliculus (SC), at the roof of the midbrain, is an evolutionarily old structure with strong commonalities across mammals, including cat, monkey, tree shrew, rat, and mouse. SC is highly interconnected with much of the brain, including the cerebellum, thalamus, hypothalamus, and neocortex, and is implicated in the coordination of several “higher-level” functions including attention and decision making. SC, however, is also an important target for sensory pathways, and sends outputs towards the motor pools, including the brainstem and spinal cord, encouraging the view that its major role may be to support rapid sensorimotor behaviours.

Substantial recent work in mouse has explored the contribution of SC to behaviour, and the functional organisation of the circuits that may support these behaviours. This work has exploited new techniques for identifying, recording, manipulating, and studying the connectivity of SC, and areas that are connected to it. The purpose of this review is to both collate this recent work and to synthesise it. The themes we will touch on are likely to be common across species, but a comparative analysis is beyond our scope, and we direct the reader to excellent recent reviews (May, 2006; Basso et al., 2021; Isa et al., 2021). Similarly, while mouse SC has been shown to also be involved in higher-level functions, we focus on simpler behaviours because they have been the focus of most recent work, and have proved useful in starting to link structure to function.

**FIGURE 1:**
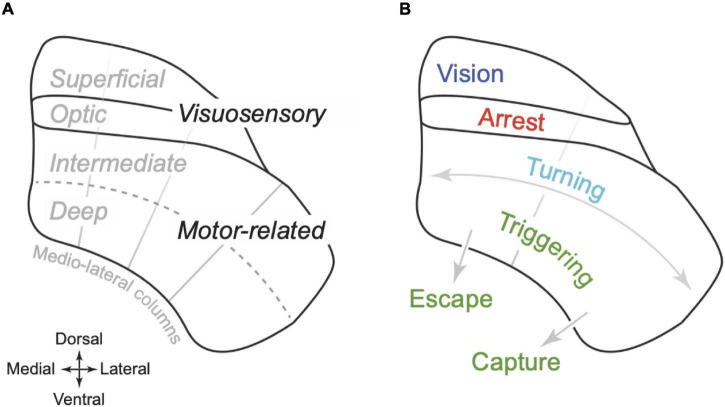
Organisation of superior colliculus in mouse. **(A)** Schematic coronal section illustrating the dorso-ventral organisation of the superior colliculus (SC) of mouse. The optic layer is ventral to the superficial layers (which includes the superficial grey and zonal layer). Together these layers are termed the “visuosensory SC.” Ventral to the optic layer is the intermediate and then the deep layer. Together these layers are termed “motor-related SC.” The radial grey lines indicate an approximate division of the SC into four “columns” that extend across the layers, partitioning SC on the medial-lateral axis (Benavidez et al., 2021). **(B)** Proposed functional organisation of SC. The superficial layers primarily support visual analysis; the optic layers primarily organise arrest behaviours; the motor-related SC supports egocentric turning movements as well as the triggering of more complex behaviours, either towards objects including prey (“capture,” lateral SC) or towards refuge (“escape,” medial SC). Turning and triggering may be primarily supported by the intermediate and deep layers, respectively.

Anatomical sections through mouse SC reveal horizontal layers with distinct cellular, and histochemical organisation (Puelles et al., 2012; Figure 1A). We will use the term “visuosensory SC” to define the layers closest to the dorsal surface, which comprise the “optic” layer as well as the “supraoptic” layers dorsal to it (superficial grey and zonal layers; Dong, 2008). Ventral to the optic layer are the “intermediate” and then “deep” layers, which can be collectively termed the “motor-related SC” (Dong, 2008). Recent work suggests that the medial-lateral axis of SC can also be parcellated, into four columns that extend across layers (Benavidez et al., 2021). These columns are defined by the patterns of inputs and outputs and as yet have no known histochemical correlates.

**FIGURE 2:**
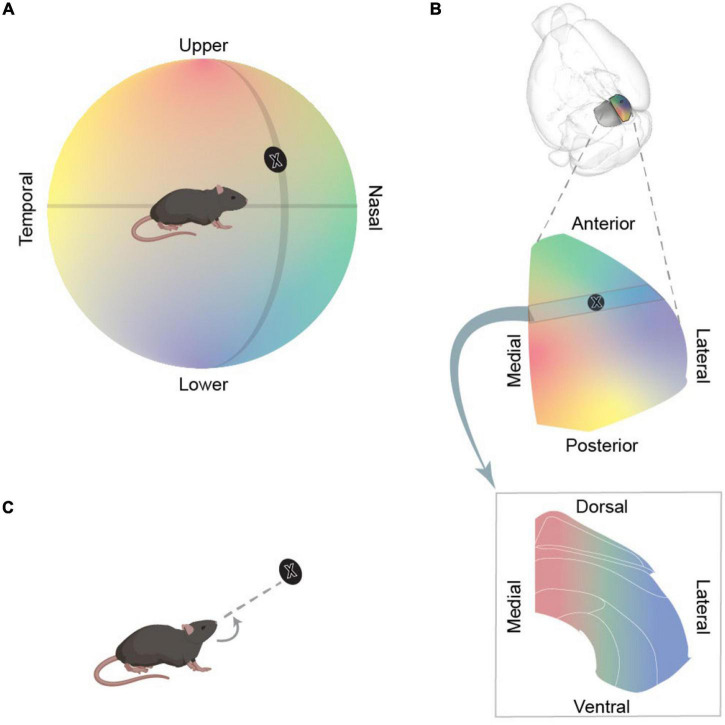
Topographic organisation of SC. **(A)** Schematic illustrating a mouse, and the world to its left, as mapped onto a hemisphere around the mouse. The direction of objects are defined in terms of visual angle: their azimuth (position along the nasal-temporal axis) and their elevation (position along axis from the upper-lower field). Azimuth and elevation axes are depicted as black lines and the black disc represents an example object. **(B)** Schematic representation of the mapping of the world onto different sections of SC. *Top panel* illustrates the position of SC in the mouse brain. *Middle panel* enlarges the right SC and shows how azimuth and elevation axes of visual field are mapped onto SC. The black disc indicates the approximate location of object in panel **(A)** in this map. *Bottom panel* illustrates the mapping of the world onto a coronal section through SC. **(C)** Activation of intermediate layer would evoke a turn towards the egocentric location represented by that region of SC.

The main sensory input to “visuosensory SC” is visual. Indeed, in mice, SC is the primary target of the retina (Ellis et al., 2016), and this input is supplemented by extensive projections from visual cortex. Visuosensory SC mainly represents the contralateral visual field, with nasal-to-temporal azimuthal axis of the visual field mapped onto the anterior-to-posterior axis of SC, and lower-to-higher elevation axis of the visual field mapped onto the lateral-to-medial axis of SC (Xu et al., 2011; Figures 2A,B). For example, the anterior-lateral SC is activated by objects in front of the animal, below the eye, and the posterior-medial SC is activated by objects behind the animal, above the eye (e.g., Mrsic-Flogel et al., 2005). Neurons in the “motor-related” parts of SC can respond to visual stimuli, but also to other sensory modalities, receiving subcortical facial somatosensory and auditory input from the trigeminal nuclei and the inferior colliculus, respectively (e.g., Benavidez et al., 2021). The topographic map of visual space found in the visuosensory SC is impressively matched to the maps of auditory (at least for the azimuthal axis) (Ito et al., 2020) and somatosensory (Drager and Hubel, 1975) space in the motor-related SC.

## The Proposed Functional Organisation of Superior Colliculus

As we will describe, activation of neurons in the mouse SC can evoke varied behavioural responses. Some of these behavioural responses are relatively simple and well described. First, some activations cause a cessation of movement, a stoppage we will call “arrest.” Second, some activations produce fixed rotational movements of the eye, tongue, head or of the whole body. We will call these egocentric rotations “turning” (Figure 2C). Third, some activations induce more complex behaviours, such that SC appears to provide a trigger, an impetus, or opens a gate for actions designed to achieve a certain outcome^1^. In some cases, this movement is towards an appetitive object (including prey), and we will call these actions “capture.^2^” In other cases, this movement is towards a place of apparent refuge, an action that we will call “escape^3^.”

An influential framework for understanding the functional organisation of the rodent SC was provided by Dean et al. (1989), synthesising a large body of work in rat. The core of this framework was a subdivision into medial and lateral SC. The lateral subdivision (which would approximately correspond to the lateral two columns in mouse SC), which represents a sensory stimulus in the lower parts of the contralateral visual field, provided outputs that “crossed” hemispheres, and appeared to be involved in turning and approach towards that sensory stimulus. The medial subdivision (or medial two columns), which represents a sensory stimulus in the upper parts of the visual field, provided uncrossed outputs and appeared to be involved in behaviours, including arrest or fast locomotion, that facilitate avoidance of potential threats.

Here we ask if a modified framework for the organisation of SC can explain new data in mouse. We hypothesise an organisational framework where: (1) Arrest is subserved by circuitry in the optic layer of SC; (2) Contralateral turning movements are likely to be subserved by circuitry in the intermediate layers that span lateral and medial SC; and (3) Deep layers of SC appear to be involved in triggering more complex behaviours including capture (lateral SC) and escape (medial SC) (Figure 1B). While manipulations of SC are sufficient to generate these behaviours, SC is likely only one part of a network of brain areas involved. Indeed, a hallmark of SC is the fact that so many areas of the brain give input to, or receive output from it. In the following sections, we therefore describe evidence for the hypothesised framework from direct manipulations of SC, from the topography of SC’s sensorimotor function, and from SC’s anatomical connectivity.

## How Manipulations of Superior Colliculus Effect Behaviour

In the following sections we review how manipulations of specific neural populations in SC lead to distinct behaviours, and note in advance that effects of such manipulations need to be treated with caution (reviewed in Jazayeri and Afraz, 2017; Wolff and Ölveczky, 2018).

### Arrest

Arrest is often thought of as an avoidance response, and is called freezing when the mouse is in a context with a potential threat. However, arrest may also be part of more exploratory behaviours, allowing for a pause for surveillance, or attention to the external environment (Botta et al., 2020). Direct manipulations of SC support the idea that the optic layer is important in arrest. Activation (Sans-Dublanc et al., 2021) or inhibition (Xie et al., 2021) of neurons concentrated in the optic layer induces or impairs arrest, respectively. Other manipulations of SC that evoke arrest have either included (Wei et al., 2015), or have focussed on (Zingg et al., 2017), the optic layer. Manipulation of CAMK2+ SC neurons at the border of the optic layer and intermediate layers can also elicit arrest behaviour (Wei et al., 2015; Cai et al., 2022). In common with some other neurons in the optic layer, these neurons appear to project to the lateral posterior nucleus of the thalamus (LP) and activation of their terminals in LP evokes arrest (Wei et al., 2015). For simplicity, we therefore include these neurons as part of the optic layer.

Arrest behaviour is more likely to be elicited by activation of medial SC, than lateral SC (Wei et al., 2015). This medio-lateral asymmetry is consistent with the previously hypothesised medio-lateral separation of function (Dean et al., 1989). Our hypothesis predicts that the absence of arrest behaviours during activation of lateral SC can be explained by the fact that the optic layer does not extend into the lateral most columns of SC (Figure 1; Benavidez et al., 2021).

### Turning

It is well established that activation of the motor-related SC in freely moving animals produces contralateral turning movements, or biases an animal towards making them (Stubblefield et al., 2013; Masullo et al., 2019; Cregg et al., 2020; Essig et al., 2021). In head-fixed mice, activation of motor-related SC biases eye movements (Zahler et al., 2021) towards the contralateral side. Inhibiting motor-related SC does the opposite, biasing the animal towards ipsilateral movements (Stubblefield et al., 2013; Lee and Sabatini, 2021). We note that when head-fixed mice rotate a ball or wheel beneath them, most active neurons in motor-related SC prefer ipsilateral turns (Steinmetz et al., 2019) and unilateral inhibition of motor-related SC biases animals away from ipsilateral turns (Huda et al., 2020). The likely explanation is that these “ipsilateral” turns require mice to push the ball or wheel down on its ipsilateral side, a movement associated with contralateral turning during free behaviour (Huda et al., 2020).

The intermediate layers of SC appear to be particularly important in turning. Activation of PITX2+ neurons in SC, which are concentrated in the intermediate layers, evokes turning (Masullo et al., 2019). The intermediate (but not deep layers) are targeted by the substantia nigra pars reticulata (SNr; Lee J. et al., 2020), and activation of SNr terminals in SC induces turning (Villalobos and Basso, 2020). Likewise, the intermediate (but not deep layers) project to neurons in the gigantocellular nucleus that are important in turning (Cregg et al., 2020).

Lateral SC is known to be involved in turning, and there is some evidence for a role of medial SC in turning. Activation of lateral SC mostly evokes contralateral turning (Isa et al., 2020), while inhibition of lateral SC neurons can impair instinctive turning towards sounds (Vale et al., 2020) and other turning behaviours (Sooksawate et al., 2013; Isa et al., 2020). Circuits for turning may, however, include medial SC as well as lateral SC. Activation of PITX2+ neurons in medial SC induces head turns (Masullo et al., 2019). Activating progressively more medial locations of SC evoked larger pitch angles of head rotation, suggesting that medial SC is involved in making movements towards more elevated angles (Masullo et al., 2019). Pitch rotations of the head produced by medial SC are difficult to measure and have only been achieved in a limited number of experiments–more data would help strengthen the case for a role of medial SC in turning.

Superior colliculus is primarily concerned with turning towards the contralateral side, but activation of anterior-medial SC can also evoke ipsiversive head turns (Isa et al., 2020), and an ipsiversive bias has been reported for some medial SC neurons (cuneiform nucleus-projectors; Isa et al., 2020) but not others (primary auditory cortex-recipient; Zingg et al., 2017). Activation of inhibitory, GABAergic SC neurons can bias animals towards contralateral movements (Essig et al., 2021; Sans-Dublanc et al., 2021) or ipsilateral movements (Duan et al., 2021; Hao et al., 2021); the specific movements elicited by activating subpopulations of neurons in SC is therefore likely to depend on both the specific projection patterns and effects (excitatory, inhibitory) of those neurons.

### Escape and Capture

Circuits in medial SC are clearly important in triggering escape behaviours. Activation of medial, motor-related SC neurons can evoke escape or putatively escape-related backwards walking and fast forwards running (Zingg et al., 2017; Evans et al., 2018; Isa et al., 2020); inhibition of medial motor-related SC impairs the triggering of escape (Evans et al., 2018). By contrast, inhibition of lateral SC does not affect the production or speed of escape behaviour (Shang et al., 2019; Vale, 2020; Huang et al., 2021), though it can affect the direction of escape (Vale et al., 2020).

Circuits in lateral motor-related SC appear more important in triggering capture behaviours. Inhibition of lateral motor-related SC neurons (including those projecting to subthalamus- or substantia nigra pars compacta, SNc; Shang et al., 2019; Huang et al., 2021) impairs capture, that is movements towards prey (Shang et al., 2019; Huang et al., 2021; Xie et al., 2021), food or conspecifics (Huang et al., 2021).

Escape and capture are both complex behaviours, that involve a combination of actions, such as turns combined with locomotion. However, activations that induce escape and capture behaviours produce turns that appear goal directed, rather than the stereotypical ego-centric turns that comprise “*turning*” behaviours described above (Evans et al., 2018; Shang et al., 2019; Huang et al., 2021; Xie et al., 2021). Whether and how escape and capture behaviours recruit circuits for “*turning*” is not yet clear. Activation of PITX2+ neurons in intermediate layers of SC induces turning without locomotion (Masullo et al., 2019), but large-scale inhibition of PITX2+ neurons does appear to impair capture (Xie et al., 2021). Thus, while we hypothesise that turning is supported primarily by neurons in intermediate SC, and triggering of capture and escape is supported primarily by neurons in deep SC, direct manipulations of motor-related SC are yet to reveal the relative contribution of intermediate and deep parts of SC in these behaviours.

We hypothesise that SC plays a similar role in both capture and escape: the output of deep layer SC triggers goal-directed action. The specific goal of that action depends on whether that signal arises in the lateral (e.g., prey) or medial (e.g., refuge) subdivisions of the deep layer, because these subdivisions have different connections to the rest of the brain, as we review below. However, the computations performed by the lateral- and medial parts of deep layer SC are predicted to be the same in both cases.

### How Sensory and Motor Function are Topographically Organised in Superior Colliculus

The major sensory inputs to SC–visual, auditory and somatosensory–are organised into aligned topographic maps (Drager and Hubel, 1975; Ito et al., 2020). These maps provide a representation of the egocentric direction of an object relative to the animal’s head: the direction of an auditory or somatosensory stimulus is directly related to the head-centric direction of the object that produces them; the location of an object’s image on the retina, if eye-movements are ignored, is also a proxy for head-centric object direction. These sensory maps are aligned parallel to the surface of SC, orthogonal to the proposed laminar organisation of function. The sensory maps may therefore be important in guiding and constraining the behaviour(s) that are elicited by sensory stimuli presented at particular directions relative to the animal.

The alignment between sensory and motor maps in SC is likely to be important in turning behaviours. Visuosensory SC includes “narrow-field” cells (Gale and Murphy, 2014) that project into topographically aligned parts of motor-related SC and appear important in turning towards prey (Hoy et al., 2019). Consistently, activation of motor-related SC evokes turning towards the directions that are represented by the equivalent location in the sensory maps (Figure 2). Activation of PITX2+ neurons at specific locations in the intermediate layers evokes contralateral turns towards specific directions (Masullo et al., 2019). Activation of more posterior PITX2+ neurons evokes larger contralateral (yaw) turns, consistent with the more temporal receptive fields found in posterior SC. Activation of more medial PITX2+ neurons evokes larger pitch turns, consistent with the more elevated receptive fields found in the medial SC. Similarly, in head-fixed mice activation of more posterior SC neurons induces more temporal eye turns (Wang et al., 2015).

The relationship between topographic sensory maps and other actions (escape, capture, arrest) is less clear. Activation experiments show that motor-related medial SC is important in escape behaviour, and visuosensory medial SC represents the overhead visual field. If this topographic alignment were important for escape behaviour, then escape behaviours should be more easily elicited by stimuli in the upper visual field (cf., Dean et al., 1989), which would include aerial, and tall ground-based predators. Indeed, an expanding black disc on a screen (“looming stimulus”) presented to the upper visual field usually elicits a rapid escape to refuge when one is present (Yilmaz and Meister, 2013). Whether looming stimuli from other visual directions can induce escape responses is less clear, but limited work suggests that a looming stimulus in front (Zhou et al., 2019) or below (Yilmaz and Meister, 2013; Zhou et al., 2019) a mouse does not elicit the same rapid escape. Capture can be directed towards stimuli in the lower visual field (Hoy et al., 2016; Vale et al., 2020), and perhaps specific parts of the lower visual field (Hoy et al., 2016, 2019; Michaiel et al., 2020; Holmgren et al., 2021; Johnson et al., 2021). Whether capture behaviours can be evoked by a stimulus presented to the upper visual field remains to be seen. Behavioural work suggests that arrest can be induced by a visual stimulus presented to either the upper or lower visual field (De Franceschi et al., 2016; Procacci et al., 2020).

## How Areas Connected to Superior Colliculus Influence Behaviour

**FIGURE 3:**
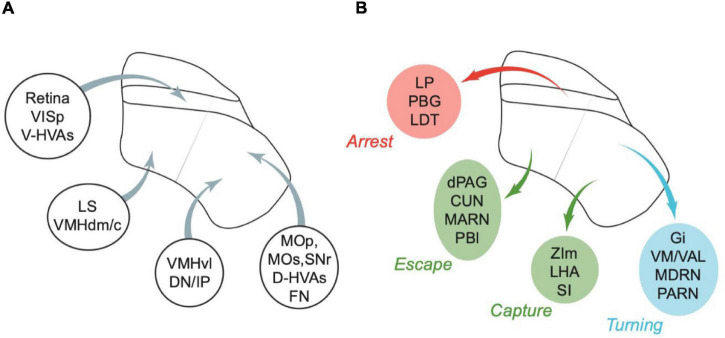
Summary of some of the major inputs and outputs of SC. **(A)** Inputs. Areas are grouped by major target regions in SC. **(B)** Outputs. Areas are grouped by major source regions in SC and proposed functional roles, indicated next to the group. Arrows in panels **(A,B)** show approximate locations of the input targets **(A)** or projection sources **(B)**. CUN, cuneiform nucleus; D-HVAs, higher visual areas (dorsal stream); DN, dentate nucleus; dPAG, dorsal PAG; FN, fastigial nucleus; Gi, gigantocellular nucleus; IP, interposed nucleus; LDT, laterodorsal tegmental nucleus; LHA, lateral hypothalamus; LP, lateral posterior nucleus of the thalamus; LS, lateral septal nucleus; MARN, magnocellular reticular nucleus; MDRN, medullary reticular nucleus; MOp, primary motor area; MOs, secondary motor area; PARN, parvicellular reticular nucleus; PBG, parabigeminal nucleus; PBl, lateral parabrachial nucleus; SI, substantia innominata; SNr, substantia nigra (reticular part); VAL, ventral anterior-lateral complex of the thalamus; V-HVAs, higher visual areas (ventral stream); VISp, primary visual area; VM, ventral medial nucleus of the thalamus; VMHdm/c, ventromedial hypothalamic nucleus (dorsomedial/central part); VMHvl, ventromedial hypothalamic nucleus (ventrolateral part); ZIm, zona incerta (medial part). Abbreviations are also defined in Table 6. Appropriate references can be found in the text and in Tables 1–5.

Connections of different parts of SC with other brain areas provides complementary, circumstantial evidence for the parcellation of behavioural function proposed in Figure 1B. This evidence is summarised below and in Figure 3. Tables 1–5 summarise the key experimental methods and results of these studies as look-up tables, and direct the reader to additional related work.

**TABLE 1:** Areas reported to be involved in the production of arrest behaviour.

ARREST											
AREA	References	Function				Anatomy					
		Neural activity increases during arrest	Activation induces arrest	Inhibition impairs arrest	Other roles	Output from ipsilateral SC			Input to ipsilateral SC		
						Layers and columns of SC	Cellular markers in SC	Cellular markers in target area	Layers and columns of SC	Cellular markers in SC	Cellular markers in source area
PBG	Benavidez et al., 2021					SCs (c. 1–3)					
	Gale and Murphy, 2018						GRP+, GAD2+ or RORB+				
	Montardy et al., 2021						DRD2+				
	Shang et al., 2018	VGLUT2+	VGLUT2+ (unilateral)	VGLUT2+ (bilateral)	Fast locomotion		PV+				
	Tokuoka et al., 2020								SCs (BILATERAL)		CHAT+
	Zhang et al., 2019						VGAT+				
	Zingg et al., 2017						Retina- or VISp- or AUDp-recipient				
LDT	Benavidez et al., 2021					SCm (c. 1–3)					
	Wang et al., 2019d							PV+ or SOM+			
	Xie et al., 2021						CBLN2+				
	Yang et al., 2016		PV+ (unilateral)	PV+ (unilateral)	SOM+ in opposing arrest						
l/vlPAG	Tovote et al., 2016		VGLUT2+ (bilateral)	VGLUT2+ (bilateral)							
	Vaaga et al., 2020		CHX10+ (bilateral)								
	Yu et al., 2021		VGLUT2+ (bilateral)								
LP	Benavidez et al., 2021					SCs, SCm (c. 1–3) (BILATERAL)					
	Gale and Murphy, 2014						NTSR1+				
	Montardy et al., 2021						DRD2 +				
	Shang et al., 2018	VGLUT2+	VGLUT2+ (unilateral)	VGLUT2+ (bilateral)			PV +				
	Wei et al., 2015						CAMK2+				
	Xie et al., 2021						CBLN2+				
	Zhou et al., 2017						SP+				
	Zingg et al., 2017						Retina- or VISp-recipient				
VISp	Liang et al., 2015		RPB4+ (bilateral)	General population							

*Functional measurements and anatomical connectivity with SC. For example, Shang et al. (2018) (4th line in PBG above) shows that; VGLUT2+ PBG neurons show increased activity during arrest; unilateral activation of VGLUT2+ PBG neurons induces arrest; bilateral inhibition of VGLUT2+ PBG neurons impairs arrest. This does not imply that these functional observations would hold in all contexts, nor that VGLUT2+ PBG neurons are the only cell type involved in arrest, nor that all VGLUT2+ PBG neurons are involved in arrest. The study also finds that PV+ SC neurons project to the PBG: though this does not imply that PV+ SC neurons are the only PBG-projectors in the SC, or that all PV+ SC neurons project to the PBG. Finally, the study further suggests a role for the PBG in fast locomotion.*

**TABLE 2:** Areas reported to be involved in the production of turning.

TURNING											
AREA	References	Function				Anatomy					
		Neural activity increased during turns	Unilateral activation biases turning	Unilateral inhibition biases turning	Other roles	Output from ipsilateral SC			Input to ipsilateral SC		
						Layers and columns of SC	Cellular markers in SC	Cellular markers in target area	Layers and columns of SC	Cellular markers in SC	Cellular markers in source area
**HEAD OR BODY**											
Gi	Benavidez et al., 2021					SCm (c. 3–4) (CONTRALATERAL)					
	Cregg et al., 2020		CHX10+	CHX10+		SCm (c. 3–4) (CONTRALATERAL)	Intermediate layer, VGLUT2+	CHX10+			
	Duan et al., 2021						MOs-recipient				
	Usseglio et al., 2020		CHX10+	CHX10+							
VTA	Barbano et al., 2020				Escape						
	Benavidez et al., 2021					SCm (c. 1–4)					
	Hughes et al., 2019	VGAT+	VGAT+	VGAT+	Not escape						
	Zhang et al., 2019				Escape		GAD2+ or VGAT+	TH+			
	Zhou et al., 2019						CAMK2+	GAD2+			
PF	Benavidez et al., 2021					SCm (c. 1–4)					
	Watson et al., 2021	General population	VGLUT2+	VGLUT2+							
STN	Benavidez et al., 2021					SCm (c. 3–4)					
	Guillaumin et al., 2021	General population	PITX2+	PITX2+							
**TONGUE OR EYE**											
MOs, ALM, FN	Benavidez et al., 2021								SCm (c. 3–4)		
	Duan et al., 2021			MOs							
	Gao et al., 2018	FN	FN	FN							
	Guo et al., 2014			ALM							
	Itokazu et al., 2018	MOs	MOs	MOs							
	Masullo et al., 2019									PITX2+	
VM/VAL	Benavidez et al., 2021					SCm (c. 3–4)					
	Guo et al., 2017			General population							
	Zingg et al., 2017						VM: MOp-recipient				
MOp	Benavidez et al., 2021								SCm (c. 3–4)		
	Masullo et al., 2019									PITX2+	
	Mayrhofer et al., 2019	MOp Tongue-jaw region		MOp Tongue-jaw region							
D-HVAs	Garrett et al., 2014									Lateral	
	Itokazu et al., 2018	RL, A									
	Odoemene et al., 2018			AM							
	Wang and Burkhalter, 2013									Intermediate layers	
**FORELIMB**											
MOp	Benavidez et al., 2021								SCm (c. 3–4)		
	Masullo et al., 2019										PITX2+
	Heindorf et al., 2018	MOp Forelimb region									
	Hira et al., 2015		MOp Forelimb region								
	Morandell and Huber, 2017			MOp Forelimb region							
MDRNv	Benavidez et al., 2021					SCm (c. 3–4)					
	Esposito et al., 2014			VGLUT2+ (bilateral)		SCm (c. 3–4) (CONTRALATERAL)	VGLUT2+	VGLUT2+			
	Masullo et al., 2019						PITX2+				
PARN, SPVO/I	Benavidez et al., 2021					SCm (c. 3–4) (4 for SPV)			SCm (c. 4) (SPV)		
	Ruder et al., 2021	General population	General population	(bilateral)							
	Han et al., 2017				PARN–oromotor						

*Functional measurements and anatomical connectivity with SC. Conventions as in Table 1.*

**TABLE 3:** Areas reported to be involved in the production of fast locomotion.

FAST LOCOMOTION									
AREA	References	Function				Anatomy			
		Neural activity increased during behaviour	Unilateral activation induces behaviour	Bilateral inhibition impairs behaviour	Other roles	Output from ipsilateral SC			Input to ipsilateral SC
						Layers and columns of SC	Cellular markers in SC	Cellular markers in target area	Layers and columns of SC
AUDp	Li et al., 2021			General population	Arrest				
	Xiong et al., 2015		RPB4+	General population					
CUN	Benavidez et al., 2021					SCm (c. 1)			
	Caggiano et al., 2018		VGLUT2+	VGLUT2+					
MARN	Benavidez et al., 2021					SCm (c. 1–2)			
	Capelli et al., 2017		LPGi VGLUT2+	LPGi VGLUT2+	LPGi VGAT+ in arrest			LPGi VGLUT2+	
IC	Benavidez et al., 2021					SCm (c. 1–4)			SCm (c. 1–4)
	Masullo et al., 2019						PITX2+		
	Xiong et al., 2015		CAMK2+	General population					
	Zhang et al., 2019						VGAT+		

*Functional measurements and anatomical connectivity with SC. Conventions as in Table 1.*

**TABLE 4:** Areas reported to be involved in the triggering of capture and escape.

TRIGGERING											
AREA	References	Function				Anatomy					
		Neural activity increased during behaviour	Unilateral activation induces behaviour	Bilateral inhibition impairs behaviour	Other roles	Output from ipsilateral SC			Input to ipsilateral SC		
						Layers and columns of SC	Cellular markers in SC	Cellular markers in target area	Layers and columns of SC	Cellular markers in SC	Cellular markers in source area
**CAPTURE**											
LHA	Benavidez et al., 2021					SCm (c. 3–4)					
	Li Y. et al., 2018	VGAT+	VGAT+ (bilateral)	VGAT+	VGLUT2+ in evasion						
	Venner et al., 2019							VGAT+			
SI	Benavidez et al., 2021								SCm (c. 4)		
	Zhu et al., 2021	THY1+	THY1+ or CAMK2+	THY1+							
l/vlPAG	Yu et al., 2021	General population		VGAT+							
VISp	Burgess et al., 2017			General population							
VMHvl	Benavidez et al., 2021								SCm (c. 1–4) (BILATERAL)	Lateral-biased	
	Wang et al., 2019b	ESR1+			Social defence						
	Lee et al., 2014		ESR1+	ESR1+							
ZIm	Ahmadlou et al., 2021		GAD2+ or TAC1+	GAD2+ or TAC1+							
	Benavidez et al., 2021					SCm (c. 4)			SCm (c. 4)		
	Masullo et al., 2019						PITX2+				
	Wang et al., 2019c				Defence						
	Xie et al., 2021						PITX2+				
	Zhao et al., 2019		VGAT+ (bilateral)	VGAT+							
MRN	Benavidez et al., 2021					SCm (c. 3–4)			SCm (c. 3–4)		
	Inagaki et al., 2020			Thalamus-projecting MRN/PPN							
	Masullo et al., 2019						PITX2+			PITX2+	
DN and IPN	Benavidez et al., 2021								SCm (c. 3–4)		
	Dacre et al., 2021		General population								
	Masullo et al., 2019									PITX2+	
AM and VAL	Benavidez et al., 2021					SCm (c. 3–4)					
	Dacre et al., 2021										
**ESCAPE**											
dPAG	Deng et al., 2016	General population	CAMK2+		Interspersed with arrest						
	Evans et al., 2018	VGLUT2+	VGLUT2+ (bilateral)	VGLUT2+			Medial-biased				
	Kunwar et al., 2015		VGLUT2+		Interspersed with arrest						
	Tovote et al., 2016		VGLUT2+		Interspersed with arrest						
PBl	Benavidez et al., 2021					SCm (c. 1)					
	Han et al., 2015	CGRP+		CGRP+	Arrest						
	Sun et al., 2020		VGLUT2+, CAMK2+								
LS	Benavidez et al., 2021								SCm (c. 1)		
	Azevedo et al., 2020	NTS+									
VMHdm/c	Benavidez et al., 2021								SCm (c. 1–4) (BILATERAL)	Medial-biased	
	Kunwar et al., 2015		SF1+ (bilateral)		Arrest						

*Functional measurements and anatomical connectivity with SC. Conventions as in Table 1.*

**TABLE 5:** Areas providing inhibitory input to the SC.

INHIBITORY INPUTS							
AREA	References	Function			Anatomy		
		Neural activity increased during behaviour	Unilateral activation induces behaviour	Bilateral inhibition impairs behaviour	Input to ipsilateral SC		
					Layers and columns of SC	Cellular markers in SC	Cellular markers in source area
LGv	Benavidez et al., 2021				SCm (c. 1–3)		
	Fratzl et al., 2021				All layers	Medial-biased VGAT+ or GAD2+ or VGLUT2+	
	Salay and Huberman, 2021					VGAT+ or GAD2+ or VGLUT2+	
LGv (ARREST)	Fratzl et al., 2021		VGAT+ activation impairs arrest				
	Salay and Huberman, 2021		GAD2+ impairs and VGLUT2+ induces arrest	GAD2+ facilitates and VGLUT2+ impairs arrest			
LGv (ESCAPE)	Fratzl et al., 2021		VGAT+ impairs escape	VGAT+ facilitates escape			
SNr (TURNING)	Benavidez et al., 2021				SCm (c. 1–4)		
	Hormigo et al., 2021		VGAT+ activation induces ipsiversive turns	VGAT+ inhibition induces contraversive turns			
	Lee K. H. et al., 2020				Intermediate layers		
	Liu et al., 2020						GAD2+ or PV+
	Masullo et al., 2019					PITX2+	
	McElvain et al., 2021						PV+ or VGAT+

*Functional measurements and anatomical connectivity with SC. Conventions as in Table 1.*

**TABLE 6:** Acronyms and corresponding brain areas used in the text.

Acronym	Definition
*ALM*	Anterolateral motor cortex (Komiyama et al., 2010)
*AM*	Anteromedial nucleus of the thalamus
*AUDp*	Primary auditory area
*CUN*	Cuneiform nucleus
*D-HVAs*	Higher visual areas, dorsal stream: RL, A, AM (Wang and Burkhalter, 2013)
*DN*	Dentate nucleus
*dPAG*	Dorsal PAG (Evans et al., 2018)
*FN*	Fastigial nucleus
*Gi*	Gigantocellular nucleus (Cregg et al., 2020)
*IC*	Inferior colliculus
*IP*	Interposed nucleus
*l/vlPAG*	Lateral/ventrolateral PAG (Han et al., 2017)
*LDT*	Laterodorsal tegmental nucleus
*LGv*	Ventral lateral geniculate nucleus
*LHA*	Lateral hypothalamic area
*LP*	Lateral posterior nucleus of the thalamus
*LS*	Lateral septal nucleus
*MARN*	Magnocellular reticular nucleus
*MDRNv*	Medullary reticular nucleus, ventral part
*MOp*	Primary motor area
*MOs*	Secondary motor area
*MRN*	Midbrain reticular nucleus
*PAG*	Periaqueductal grey
*PARN*	Parvicellular reticular nucleus
*PBG*	Parabigeminal nucleus
*PBl*	Lateral parabrachial nucleus (Sun et al., 2020)
*PF*	Parafascicular nucleus
*SI*	Substantia innominata
*SCs*	Visuosensory SC
*SCm*	Motor-related SC
*SNr*	Substantia nigra, reticular part
*SPVO/I*	Spinal nucleus of the trigeminal, oral, and interpolar parts
*STN*	Subthalamic nucleus
*VAL*	Ventral anterior-lateral complex of the thalamus
*V-HVAs*	Higher visual areas, ventral stream: LM, LI, P, and POR (Wang and Burkhalter, 2013)
*VISp*	Primary visual area
*VM*	Ventral medial nucleus of the thalamus
*VMHdm/c*	Ventromedial hypothalamic nucleus, dorsomedial/central part (Kunwar et al., 2015)
*VMHvl*	Ventromedial hypothalamic nucleus, ventrolateral part (Lee et al., 2014)
*VTA*	Ventral tegmental area
*ZIm*	Zona incerta, medial part (Zhao et al., 2019)

*Acronyms and nomenclature are according to that used by the Allen Brain Institute, unless otherwise indicated by an associated citation.*

### Visuosensory Superior Colliculus, Arrest

Connections of visuosensory SC, and the optic layer in particular, are consistent with a role in arrest. The thalamic area LP and pontine area laterodorsal tegmental nucleus are innervated by the optic layer (Xie et al., 2021), and are involved in arrest (Yang et al., 2016; Shang et al., 2018). Midbrain area parabigeminal nucleus (PBG) has reciprocal connections with visuosensory SC (including the optic layer; Zhang et al., 2019; Tokuoka et al., 2020), and may be involved in the production of arrest following escape (Shang et al., 2018). Primary visual cortex innervates the visuosensory SC, including the optic layer, and activation of these terminals in SC (Liang et al., 2015) or of SC neurons post-synaptic to them (Zingg et al., 2017) induces arrest, while inhibition of primary visual cortex impairs arrest to light flashes (Liang et al., 2015). Interestingly, ventral stream higher visual areas primarily project to optic layer (Wang and Burkhalter, 2013) but whether they also have a role in arrest is not yet known. By contrast, while inhibition of primary auditory cortex also impairs sound-induced arrest (Li et al., 2021), primary auditory cortex’s projection to SC does not innervate the optic layer (Benavidez et al., 2021), and inhibition of SC does not impair these sound-induced arrest behaviours. The stimulus selectivity of visuosensory SC is generally broad (e.g., Gale and Murphy, 2014; De Franceschi and Solomon, 2018), and the sensory signals in these layers are therefore likely able to guide many or even most behaviours. Particular pathways through visuosensory SC may nevertheless be more important for some behaviours than others (e.g., Reinhard et al., 2019); whether projections from visuosensory to motor-related SC are particularly important for arrest, remains to be determined.

Central amygdala (CeA) is an end target of many of the pathways that project from the optic layer of SC. LP is indirectly connected to CeA through the basolateral amygdala (Fadok et al., 2018), and PBG projects directly to CeA (Shang et al., 2015). Activation of medial SC populations concentrated in (NTSR1+) (Gale and Murphy, 2014), or including (CAMK2+) (Wei et al., 2015), the optic layer activates CeA and induces arrest (Sans-Dublanc et al., 2021), and manipulating CeA alters freezing responses to visual looming stimuli (Zelikowsky et al., 2018). CeA may promote arrest or freezing *via* several potential pathways, perhaps even through its projection to periaqueductal grey (PAG) (Tovote et al., 2015; Vaaga et al., 2020; Yu et al., 2021).

### Intermediate Layers of Superior Colliculus, Turning

Intermediate layers of SC are likely to be particularly important in turning. CHX10+ gigantocellular nucleus neurons, which are a potential route through which lateral SC promotes contralateral turning, receive input from intermediate layers (but not deep layers) of SC (Cregg et al., 2020). Intermediate layers (but not deep layers) are innervated by the substantia nigra pars reticulata (Lee J. et al., 2020), and manipulation of that input induces turning (Villalobos and Basso, 2020). Dorsal stream higher visual areas, which may play a role in representing turn directions (Itokazu et al., 2018; Odoemene et al., 2018), specifically target intermediate layers (Wang and Burkhalter, 2013).

Areas involved in turning appear to preferentially connect to lateral SC. Many of the connections of lateral SC (Benavidez et al., 2021) are known to have a role in producing movements towards particular egocentric directions, either of the body (including gigantocellular nucleus; Cregg et al., 2020) or its parts (motor cortex; fastigial nucleus of the cerebellum; motor thalamus; Guo et al., 2014, 2017; Hira et al., 2015; Morandell and Huber, 2017; Gao et al., 2018; Heindorf et al., 2018; Mayrhofer et al., 2019). Lateral SC also provides output to the medullary reticular nucleus (Esposito et al., 2014) that may be important in movements of the forelimb contralateral to SC (Ruder et al., 2021), and to the parvicellular reticular nucleus that may also be important in forelimb (Ruder et al., 2021) and tongue-jaw movements (Han et al., 2017).

While there is good evidence that lateral SC is involved in turning movements, PITX2+ SC neurons are found in medial- as well as lateral SC (Masullo et al., 2019), and these neurons are known to be involved in turning. The connection pattern of PITX2+ neurons is similar to that found for non-specific tracing from lateral SC (Masullo et al., 2019; Xie et al., 2021). This suggests that while areas involved in turning have stronger connections with lateral SC, they are also connected to medial SC.

Superior colliculus also sends projections to basal ganglia nuclei, including ventral tegmental area (VTA; e.g., Zhang et al., 2019; Zhou et al., 2019) and the subthalamic nucleus. Whether these SC projections help generate specific turning actions, or more complex behaviours, is not yet clear (Hughes et al., 2019; Zhou et al., 2019; Barbano et al., 2020).

### Deep Layers of Superior Colliculus, Capture and Escape

Deep layers of SC are connected to areas that are thought to be important in triggering more complex movements. Deep lateral SC is connected to areas (Benavidez et al., 2021) involved in triggering capture [including zona incerta, medial part; substantia innominata; ventromedial hypothalamus, ventrolateral part (Lee et al., 2014; Zhao et al., 2019; Zhu et al., 2021)]; and activation of SC terminals in the zona incerta (Shang et al., 2019; Xie et al., 2021) or substantia nigra pars compacta (SNc; Huang et al., 2021), facilitates capture. Lateral SC also projects to the lateral hypothalamic area (Venner et al., 2019; Benavidez et al., 2021), also potentially involved in capture (Li Y. et al., 2018). Lateral SC receives input from the dentate and interposed nuclei of the cerebellum, areas which might be involved in triggering goal-directed movements of the forelimb (Dacre et al., 2021) and other body parts.

Deep medial SC is connected to areas involved in evoking fast locomotion (including cuneiform nucleus and magnocellular reticular nucleus; Capelli et al., 2017; Caggiano et al., 2018) and triggering escape (including dorsal periaqueductal grey, dPAG; lateral parabrachial nucleus; lateral septum; ventromedial hypothalamus, dorsomedial/central part, VMHdm/c; Han et al., 2015; Kunwar et al., 2015; Deng et al., 2016; Tovote et al., 2016; Evans et al., 2018; Azevedo et al., 2020; Sun et al., 2020). Activation of terminals of SC neurons in the PAG evokes mild running in head-fixed mice (Wang et al., 2019a) and “wild running or backward fleeing behaviours” in freely moving mice (Wei et al., 2015). Some medial SC neurons project to both cuneiform nucleus and dPAG (Isa et al., 2020).

Whether triggering of behaviour is the preserve of deep layers, or also involves intermediate layers is not yet clear. Capture-associated connections (zona incerta, medial part and ventromedial hypothalamus, ventrolateral part) also contact intermediate layers (Benavidez et al., 2021). In the case of escape, DRD2+ and PITX2+ neurons both have patchy labelling in intermediate layers of SC, but have different projections, and activation of the former can trigger escape, suggesting that some intermediate layer neurons are involved in triggering escape (Masullo et al., 2019; Montardy et al., 2021; Xie et al., 2021). However, deep layers of SC alone are innervated by VMHdm/c (Benavidez et al., 2021), and dPAG also gets more input from the deep layers of SC than intermediate layers (Evans et al., 2018). VMHdm/c and dPAG have roles in triggering escape behaviour, so their connection to the deep rather than intermediate SC would suggest the deep layers are more important in triggering these behaviours.

## Conclusion

In summary, we propose that many of the recent observations made while investigating of the role of mouse SC in simple behaviours can be explained by supposing that: (1) The optic layer is important in arrest; (2) The intermediate layers are important in turning; (3) The deep layers are involved in the triggering of more complex behaviours including capture and escape. Our hypothesis has the advantage that it predicts that the circuitry in each of the optic, intermediate and deep layers has a simple computational purpose. The proposed organisation allows homogenous organisation and expression of genetic markers within a layer, and allows homogenous circuitry and function within each layer. That is, each layer performs a particular computation, but the functional consequence of that computation depends on the particular pattern of inputs and outputs at different locations (e.g., medial or lateral) within the layer. The proposed organisation of SC is therefore similar in concept to the idea of columnar or “canonical” microcircuitry thought to be important in the function of the cerebral cortex (e.g., Miller, 2016).

Dean et al. (1989) proposed that the crossed pathway of SC (spanning the medial-lateral axis, but concentrated in lateral SC) was associated with contralaterally directed movements, whilst the uncrossed pathway (concentrated in medial SC) was associated with defensive behaviours, such as freezing, escape and ipsilaterally directed movements. We also propose that neurons promoting contralaterally directed movements are distributed across the medial-lateral axis of SC, and that escape is the preserve of medial SC, although we further hypothesise that turning and escape are associated with the intermediate and deep layers, respectively. The major differences between our proposed organisation, and that of Dean et al. (1989), is that in our organisation: arrest (including freezing) is primarily supported by neurons in the optic layer. In addition, we propose that equivalent circuitry within lateral and medial parts of the deep layers supports both capture and escape–different behaviours are triggered by medial and lateral SC because each region has distinct pattern of connections with other brain areas.

Lamprey, fish and flies turn away from threatening stimuli, and in lamprey and fish, this action is supported by ipsiversive movement-promoting neurons in homologues of SC (Isa et al., 2021). Instead, mice turn towards a refuge (when present) when they are confronted by imminent threats (Evans et al., 2018). Our proposal does not include a role for ipsiversive movement-promoting neurons in mouse SC. If correct, we speculate that this species difference may be part of a general co-option of SC’s turning circuitry in mammals, allowing mice to turn towards memorised, predicted or learned directions. These behaviours may be supported by inputs from evolutionarily newer areas in the telencephalon, including the retrosplenial and frontal cortices, and the basal ganglia. The role of these inputs would be to override the “turn towards stimuli” contingency normally represented by turning circuitry in SC, including functionally inhibiting contraversive turn-promoting neurons (c.f., Huda et al., 2020; Duan et al., 2021; Lee and Sabatini, 2021).

There remain many missing pieces that may provide substantial challenges to the proposed organisation. For example, we predict that separate neurons are involved in turning and capture, and that they are associated with the intermediate and deep layers, respectively, but there is mixed evidence for the laminar segregation of these neurons. In some SC targets (zona incerta, lateral/ventrolateral PAG, VTA), different neurons are involved in different behaviours, but whether these are appropriately connected to relevant SC neurons is untested. We have not considered the role of the extensive interhemispheric connections of SC. We also predict that visual stimuli will elicit rapid escape only if they are in the upper visual field, but there is very little data on the influence of stimulus location on escape. Finally, emerging work has now started to explore SC’s role in stimulus discrimination tasks in mouse (e.g., Stubblefield et al., 2013; Hu et al., 2019; Wang et al., 2020, 2021; Duan et al., 2021; Essig et al., 2021; Hu and Dan, 2022). How the behaviours we have focused on (arrest, capture, escape) contribute to these tasks is not yet clear. Combining the formalism of classical discrimination tasks, and the ecological relevance of the behaviours we have generally discussed here, is likely to be a fruitful direction for future research.

Much remains to be understood about the specific contribution of SC to even simple behaviours. For example, consider two potential representations provided by intermediate layers of SC, which are likely to support turning behaviours. In one scenario, intermediate layers represent the direction in which behaviour should be expressed, and other brain areas are responsible for selecting the specific behaviour that should be produced, such as choosing between orienting the eyes, head or tongue. Alternatively, the pattern of activity over SC neurons might define both direction and specific behaviour produced, similar to the action-selection model of basal ganglia function (Friend and Kravitz, 2014). Indeed, some targets of SC appear to be involved in specific types of turns (e.g., Takatoh et al., 2021). Retrograde tracing experiments also suggest that different targets of SC receive input from different SC neurons: LP-projectors are separate from PBG-projectors (Shang et al., 2018) and SNc-projectors (Huang et al., 2021); zona incerta-projectors are separate from PAG-projectors, midbrain locomotor region-projectors (Shang et al., 2019), and SNc-projectors (Huang et al., 2021); SNc-projectors are separate from VTA-projectors (Huang et al., 2021). Functional evidence is, however, limited, and it remains possible that individual SC neurons are involved in multiple aspects of turning. For example, PITX2+ SC neurons promote orienting in freely moving mice, but eye turns in head-fixed mice (Masullo et al., 2019).

Resolution of these outstanding questions is likely to be helped by the development of mouse lines in which genetically defined populations can be studied. Recent work has already provided lines which allow the targeting of neurons in different layers, often with different connections. Different zonal and upper superficial grey layer neurons can be targeted using expression of DRD1 (Montardy et al., 2021), and combinations of GAD2 and RORB (Gale and Murphy, 2018). Another population of visuosensory neurons is labelled by GRP (Gale and Murphy, 2014). In the lower superficial grey and optic layer, neurons can be targeted using expression of PV (Shang et al., 2015), NSTR1 (Gale and Murphy, 2014), CAMK2 (Wei et al., 2015), CBLN2 (Xie et al., 2021), SP (Zhou et al., 2017), and DRD2 (Montardy et al., 2021). The intermediate layers can be targeted with PITX2 (Masullo et al., 2019; Xie et al., 2021) and DRD2 (Montardy et al., 2021) expression. Cell types in the deep layers of SC, which have been defined on the basis of morphological and intrinsic electrophysiological properties, currently lack equivalent genetic markers (Bednárová et al., 2018).

Our proposal may provide a natural framework for more general understanding of the function of SC. First, threat imminence theory proposes that animals switch from freezing to escape behaviour as a threat becomes more imminent (Perusini and Fanselow, 2015). While many stimuli may elicit freezing, only some should trigger escape. This is consistent with the fact that neurons in optic layers respond to a broader range of visual stimuli than do neurons in motor-related SC (Lee K. H. et al., 2020). Second, SC is generally thought to be important in mediating visual attention, at least in primates (e.g., Krauzlis et al., 2013). If attention can be similarly described in mice (Wang and Krauzlis, 2018), then optic layer SC neurons involved in arrest (whose projections include the thalamus) may be important in pausing other behaviours to allow attention, and motor-related SC neurons involved in turning may be important in directing attention to particular locations within the visual field (Wang et al., 2020, 2021). Third, the proposed compartmentalisation of function may help rapid decision making (Gold and Shadlen, 2007). Activity in each compartment could be considered evidence in favour of a behaviour, such that behaviour is executed when accumulated activity exceeds a threshold level. Indeed, a threshold applied to the accumulated activity of neurons in motor-related SC can explain triggering of escape behaviours (Evans et al., 2018). Fourth, even these simple behaviours are context dependent–for example animals usually choose to escape from a looming visual stimulus (Yilmaz and Meister, 2013), but freeze if the refuge is distant (Lecca et al., 2020) or absent (Vale et al., 2017). Functional compartmentalisation of SC would make it straightforward to bias simple behavioural choices and thereby tune behaviour to context.

## Author Contributions

TW conducted the literature research. AS and SS supervised and obtained funding. All authors conceived and wrote the review, contributed to the article, and approved the submitted version.

## Conflict of Interest

The authors declare that the research was conducted in the absence of any commercial or financial relationships that could be construed as a potential conflict of interest.

## Publisher’s Note

All claims expressed in this article are solely those of the authors and do not necessarily represent those of their affiliated organizations, or those of the publisher, the editors and the reviewers. Any product that may be evaluated in this article, or claim that may be made by its manufacturer, is not guaranteed or endorsed by the publisher.

## Footnotes

1 Triggered behaviours usually involve goal-directed movements and may include turns. But unlike “turning,” activations trigger a motor action sequence that depends on the structure of the environment, and cannot simply be explained by a stereotypical ego-centric action.

2 “Capture” has been termed “pursuit” elsewhere (e.g., Procacci and Hoy, 2019): we choose “capture” to avoid confusion with pursuit eye-movements.

3 Note that escape is considered to be an action towards a target (e.g., the refuge) rather than away from the location of a potential threat (e.g., Vale et al., 2017).
